# Host/Guest Nanostructured Photoanodes Integrated with Targeted Enhancement Strategies for Photoelectrochemical Water Splitting

**DOI:** 10.1002/advs.202103744

**Published:** 2021-11-05

**Authors:** Zhiwei Wang, Heng Zhu, Wenguang Tu, Xi Zhu, Yingfang Yao, Yong Zhou, Zhigang Zou

**Affiliations:** ^1^ School of Science and Engineering The Chinese University of Hong Kong Shenzhen Guangdong 518172 P. R. China; ^2^ Hefei National Laboratory for Physical Sciences at the Microscale School of Chemistry and Materials Science University of Science and Technology of China Hefei Anhui 230026 P. R. China; ^3^ College of Engineering and Applied Sciences Nanjing University Nanjing Jiangsu 210093 P. R. China; ^4^ Jiangsu Key Laboratory for Nano Technology National Laboratory of Solid State Microstructures Collaborative Innovation Center of Advanced Microstructures School of Physics Nanjing University Nanjing Jiangsu 210093 P. R. China

**Keywords:** host/guest, hydrogen generation, nanostructure, photoelectrodes, water splitting

## Abstract

Photoelectrochemical (PEC) hydrogen production from water splitting is a green technology that can solve the environmental and energy problems through converting solar energy into renewable hydrogen fuel. The construction of host/guest architecture in semiconductor photoanodes has proven to be an effective strategy to improve solar‐to‐fuel conversion efficiency dramatically. In host/guest photoanodes, the absorber layer is deposited onto a high‐surface‐area electron collector, resulting in a significant enhancements in light‐harvesting as well as charge collection and separation efficiency. The present review aims to summarize and highlight recent state‐of‐the‐art progresses in the architecture designing of host/guest photoanodes with integrated enhancement strategies, including i) light trapping effect; ii) optimization of conductive host scaffolds; iii) hierarchical structure engineering. The challenges and prospects for the future development of host/guest nanostructured photoanodes are also presented.

## Graphical abstract

Host/guest photoanodes consist of high‐surface‐area host scaffolds for quick electron transport and photoactive guest films for efficient visible‐light harvesting, resulting dramatically improved PEC performance. The present review aims to summarize and highlight recent state‐of‐the‐art progresses in the architecture designing of host/guest photoanodes with the integrated enhancement strategies. The challenges for the future development of host/guest photoanodes are also presented.

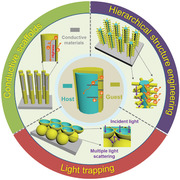

## 1 Introduction

The renewable solar energy received by the land per year (3.6 × 10^5^ Terawatts) far exceeds the predicted global energy consumption by humankind in 2050 (36 Terawatts).^[^ ^1^, ^2^, ^3^, ^4^ ^]^ Converting solar energy into renewable chemical fuels has been considered a promising solution to tackle both energy crises and environmental pollutions.^[^ ^5^, ^6^, ^7^, ^8^ ^]^ Hydrogen fuel is a clean, renewable and carbon‐free fuel, which will play a vital role in a clean, secure, and affordable energy future.^[^ ^9^, ^10^, ^11^ ^]^ Photoelectrochemical (PEC) water splitting provides a green pathway for hydrogen fuel production through the direct conversion of solar energy.^[^ ^12^, ^13^, ^14^ ^]^ In 1972, Fujishima and Honda first conducted the pioneering work for PEC water splitting via TiO_2_ films as the photoanode.^[^ ^15^ ^]^ Following that, numerous research efforts have been made to realize a high solar‐to‐hydrogen (STH) conversion efficiency.^[^ ^11^, ^12^, ^13^, ^14^ ^]^

The STH conversion efficiency of semiconductor photoanodes for practical application is primarily determined by several critical factors in the PEC process, including efficient visible light absorption, high separation and collection of charge carriers, and good long‐term chemical stability.^[^ ^16^, ^17^, ^18^ ^]^ Much effort has been made to develop advanced techniques for fabricating novel nanostructures as semiconductor photoanodes.^[^ ^19^, ^20^, ^21^ ^]^ Nanostructures can usually enhance solar energy harvesting, reduce diffusion length of the charge carriers, and increase the contact area with the electrolyte, thus improving the STH conversion efficiency of one PEC cell. Various nanostructures such as 1D nanorods (NRs)^[^ ^22^, ^23^ ^]^ and nanowires (NWs),^[^ ^24^ ^]^ 2D nanosheets (NSs),^[^ ^25^, ^26^ ^]^ 3D inverse opals (IOs)^[^ ^27^, ^28^ ^]^ have been reported for efficient PEC applications. Although these single semiconductors demonstrate high PEC performance, it is difficult to satisfy these critical factors simultaneously on a single semiconductor photoanode.^[^ ^29^, ^30^, ^31^ ^]^ Constructing multicomponent heterostructure photoanodes is one of the most effective strategies to balance the harsh elements and achieve superior properties than individual components.^[^ ^32^, ^33^, ^34^ ^]^

Inspired by the synergistic effects of a tree trunk for mass transport and leaves for light absorption in natural photosynthesis, host/guest multicomponent architectures are proposed for PEC water splitting, in which a highly dispersed light absorber acting as the guest material is deposited onto nanostructured host scaffolds.^[^ ^35^, ^36^, ^37^, ^38^ ^]^ Metal oxides such as Fe_2_O_3_^[^ ^39^ ^]^ and BiVO_4_^[^ ^40^ ^]^ are usually used as guest materials to absorb visible solar light due to the narrow bandgap and good chemical stability at the highly oxidizing condition required for water oxidation. The wide bandgap materials such as SnO_2_,^[^ ^41^ ^]^ ZnO,^[^ ^42^ ^]^ and WO_3_^[^ ^43^ ^]^ can act as host scaffolds to allow rapid electron extraction at the contact, owing to their excellent electron transport properties and suitable conduction band alignment to the guest absorber. Thus, the construction of host/guest architecture in photoanodes can simultaneously enhance visible light absorption as well as separation and collection of charge carriers, dramatically improving the solar‐to‐fuel conversion efficiency of PEC photoanodes.^[^ ^44^, ^45^ ^]^ Typically, the configuration of PEC devices can be also divided into three types, namely nonintegrated/modular, fully integrated/wireless, and partially integrated/wired PEC devices. In the nonintegrated/modular PEC devices, water splitting and light absorption are achieved by electrodes and photovoltaic cells, respectively. Conversely, for fully integrated/wireless and partially integrated/wired PEC devices, water splitting and light absorption are combined into one electrode. The anode and cathode are in physical contact in fully integrated/wireless PEC devices, while they are connected through external wiring in partially integrated/wired PEC devices.^[^ ^46^ ^]^ Mostly host/guest photoanodes are designed as nonintegrated/modular PEC devices. After decades of development, numerous host/guest photoanodes have been reported for PEC applications. Therefore, it is essential to give a timely review on the architecture progress of host/guest nanostructured photoanodes.

**Figure 1:**
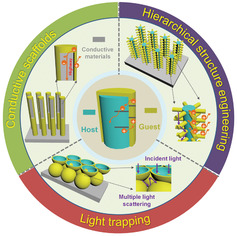
Schematic illustration of the designing architectures of host/guest nanostructured photoanodes integrated with several targeted enhancement strategies.

The present review aims to summarize and highlight recent state‐of‐the‐art progresses in the architectural design of host/guest photoanodes with integrated enhancement strategies (**Figure** **1**), including i) light trapping effect; ii) optimization of conductive host scaffolds; iii) hierarchical structure engineering. First, the principle and evaluation factors of PEC water splitting are discussed. Second, the properties of typical host or guest materials are briefly summarized. Third, the architectural progresses of host/guest photoanodes is overviewed based on the integrated enhancement strategies. Finally, the challenges and prospects for the future development of host/guest nanostructured photoanodes are also presented.

## 2 Principle and Critical Parameters of PEC Water Splitting

**Figure 2:**
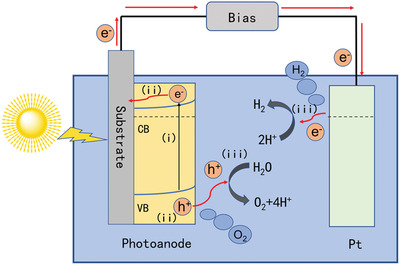
Schematic diagram of one typical PEC cell based on n‐type semiconductor photoanode.

The configuration of one typical n‐type semiconductor‐based PEC cell is shown in **Figure** **2**, consisting of a working photoelectrode for oxygen evolution reaction (OER) and a counter electrode (such as Pt) for hydrogen evolution reaction (HER). The electrodes are immersed in an electrolyte, such as neutral, acid, alkaline aqueous solutions, or sacrificial reagents. The PEC water splitting process involves three main steps. First, electron–hole pairs are generated through the absorption of incident photons with energy more prominent than the bandgap of the semiconductors. The light absorption efficiency (*η*_abs_) reflects the percentage of incident photons that are absorbed by the photoanode. Second, due to the band bending induced space‐charge field, the photogenerated electrons are transported toward the counter electrode through the external circuit and holes diffuse and migrate to the semiconductor/electrolyte interface simultaneously. Here, the separation efficiency (*η*_sep_) describes the percentage of photogenerated carriers that are successfully transported to the electrode surface. Third, the electrons or holes that transferred to the metal/electrolyte or semiconductor/electrolyte junction take part in HER or OER, respectively. The charge transfer efficiency (*η*_trans_) indicates the percentage of surface‐reaching photogenerated carriers that finally participate in the desired reactions.^[^ ^47^, ^48^, ^49^, ^50^ ^]^ Thermodynamically, water splitting is an uphill reaction with a minimum energy requirement of 1.23 eV.^[^ ^51^ ^]^ Thus, the energy of photons absorbed by a photoanode should be larger than 1.23 eV, corresponding to ≈1100 nm wavelength of light. However, the minimum practical energy required for PEC water splitting is around 1.8–2.0 eV, owing to the energy losses and the overpotential requirement for acceptable surface reaction kinetics.^[^ ^52^, ^53^ ^]^

The measured photocurrent density (*J*_pec_) at 1.23 V versus RHE is frequently selected to compare the PEC performance of different photoanodes.^[^ ^54^, ^56^ ^]^ *J*_pec_ is determined by the theoretical maximum current density (*J*_max_), *η*_abs_, *η*_sep_, and *η*_trans_, as shown in the following equations^[^ ^57^, ^58^ ^]^

(1) $$J_{\mathrm{pec}}=J_{\max}\;\times \eta_{\mathrm{abs}}\;\times \eta_{\mathrm{sep}}\times \eta_{\mathrm{trans}}$$

(2) $$J_{\max}=e\int_{0}^{1240/E_{g}}I\left(\lambda \right)\;d\lambda$$

(3) $$\eta_{\mathrm{abs}}=\frac{e\int_{0}^{1240/E_{g}}I\left(\lambda \right)\times \mathrm{LHE}\;d\lambda }{J_{\max}}$$

(4) LHE=100%−Reflectance%−Transmittance%

Where *e* is the charge of a single electron, *E*_g_ is the bandgap energy of a semiconductor, and *I*(*λ*) represents the photon flux at a different wavelength. LHE is the light‐harvesting efficiency, which can be experimentally measured using a spectrometer equipped with an integrating sphere.^[^ ^59^ ^]^

**Figure 3:**
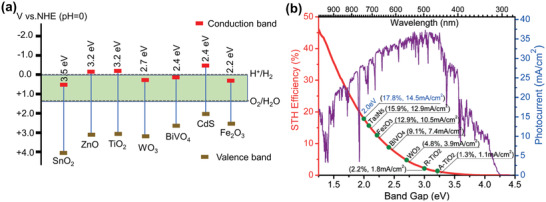
a) Schematic of bandgap positions of typical semiconductors. b) The distribution of *J*_max_ and corresponding STH efficiency over the bandgap under AM 1.5 G irradiation for different semiconductors. Reproduced with permission.^[^ ^62^ ^]^ Copyright 2015, Royal Society of Chemistry.

*J*_max_ describes the maximum photocurrent that can be achieved by a semiconductor under the assumption that all incident photons and photogenerated electrons are utilized for current formation. Bandgap energy determines the achievable *J*_max_, which is the upper limit of *J*_pec_.^[^ ^60^, ^61^ ^]^ The bandgap positions of typical semiconductors and the distribution of solar energy and *J*_max_ over the wavelength are shown in **Figure** **3**. Semiconductors with smaller bandgap energy, such as WO_3_ (2.7 eV, 3.9 mA cm^−2^), BiVO_4_ (2.4 eV, 7.4 mA cm^−2^), and Fe_2_O_3_ (2.2 eV, 10.5 mA cm^−2^) can achieve larger *J*_max_ than semiconductors with wider bandgaps, such as anatase TiO_2_ (3.2 eV, 1.1 mA cm^−2^).^[^ ^62^, ^63^ ^]^

In the practical PEC process, the measured *J*_pec_ is much lower than the theoretical *J*_max_ due to poor *η*_abs_, *η*_sep_, and *η*_trans_. Surface coating of efficient OER catalysts is a well‐established technique to improve *η*_trans_.^[^ ^63^, ^64^, ^65^ ^]^ However, the enhancement of *η*_abs_ and *η*_sep_ still remains a challenge due to their complex interaction. For example, the direct increase of film thickness is beneficial to *η*_abs_, but inevitably enlarges the carrier transfer path, resulting in low *η*_sep_.^[^ ^66^ ^]^ Therefore, the overall photocurrent of a photoanode is mainly determined by the equilibrium of *η*_abs_ and *η*_sep_. Developing rationally designed host/guest architectures with the targeted improvement strategies is a promising pathway to optimize *η*_abs_ and *η*_sep_.^[^ ^58^ ^]^ The *η*_abs_ and *η*_sep_ can be experimentally measured by using a hole scavenge, such as Na_2_SO_3_, with a *η*_trans_ of 100%.^[^ ^67^, ^68^ ^]^

When measuring photocurrent, side reactions or photocorrosion of semiconductors may also contribute to the total value of the photocurrent, resulting in the overestimation of PEC performance.^[^ ^69^ ^]^ Thus, Faradaic efficiency, incident photon‐to‐current efficiency (IPCE), and STH efficiency are frequently used as evaluation parameters. Faradaic efficiency can reveal the percentage of the charge carriers that are genuinely used for O_2_ or H_2_ production. It can be derived by dividing the electric charge calculated from the measured amount of evolved gas by the total electric charge.^[^ ^70^ ^]^ IPCE calculates the efficiency in the form of “electrons out per photons in” and takes the influences of spectral change of incident photons into consideration. IPCE describes the upper limit of efficiency to produce hydrogen or oxygen from water on the condition that all electrons and holes are used.^[^ ^52^ ^]^ STH efficiency, which is defined as the ratio of the generated chemical energy divided by the solar energy input, reports the efficiency of a PEC system under zero bias without any addition of sacrificial reagents.^[^ ^71^ ^]^ PEC system with a STH efficiency greater than 10% can be used for practical application, hence this parameter is commonly used in the ranking of a PEC device.^[^ ^72^ ^]^

## 3 Review of Host and Guest Materials

The host/guest photoanodes reported in recent years are summarized in **Table** **1**. SnO_2_, WO_3_, and ZnO are primary host scaffold materials, while narrow bandgap semiconductors, such as Fe_2_O_3_ and BiVO_4_ are typical guest materials. The architectures of nanostructured host/guest photoanodes can be divided into 1D core/shell nanostructures and hierarchical nanostructures, such as 1D/1D nanodendrite arrays,^[^ ^68^ ^]^ 1D/2D screw‐like nanostructures,^[^ ^41^ ^]^ and 3D/2D nanospheres/NSs.^[^ ^35^ ^]^ Although various architectures have been reported as host/guest photoanodes, their fabrication process can be divided into two independent parts: the growth of nanostructured host scaffolds and the following surface coating of guest semiconductor films. Thus, the benefits of one host scaffold can be applied to different guest materials. The host semiconductors usually exhibit little visible light absorption, owing to their wide bandgap. The bandgap energies of SnO_2_ and ZnO are 3.5 and 3.2 eV, respectively, making little contribution to visible light absorption in the host/guest photoanodes. WO_3_ has a bandgap of 2.7 eV that can harvest 12% of solar spectrum absorption.^[^ ^91^ ^]^ Compared to host scaffolds, guest semiconductors with narrow bandgaps have stronger visible light absorption. However, many guest semiconductors suffer from poor electron mobility. For example, Fe_2_O_3_ has low electron mobility (≈10^−2^ cm^2^ V^−1^ s^−1^) and a short hole diffusion length of 2–4 nm.^[^ ^92^, ^93^, ^94^ ^]^ Although BiVO_4_ has a moderate hole diffusion length of 100–200 nm, it is restricted by poor electron mobility (≈0.02–0.044 cm^2^ V^−1^ s^−1^).^[^ ^95^, ^96^ ^]^ Under frontside light illumination, the photoexcited electrons are generated near the photoanode/electrolyte interface and have to cross the entire film thickness for chemical reaction. Thus, the low electron mobility inevitably restricts the transport of photogenerated electrons to the electron collector. On the contrary, host semiconductors exhibit a lower visible light absorption but much higher electrons mobility than guest materials, such as SnO_2_ (≈240 cm^2^ V^−1^ s^−1^),^[^ ^97^ ^]^ ZnO (≈200 cm^2^ V^−1^ s^−1^),^[^ ^98^ ^]^ WO_3_ (≈12 cm^2^ V^−1^ s^−1^).^[^ ^99^ ^]^ The improvement of host scaffolds on the charge collection of guest materials will be detailed in Section 4.2.

**Table 1:** Representative host/guest photoanodes for PEC water splitting

Host materials	Architecture	Host/guest photoanodes	Electrolytes	Photocurrent density [mA cm^−2^] at 1.23 V versus RHE under AM 1.5 [100 mW cm^−2^]	Refs.
SnO_2_	1D/0D	Sb: SnO_2_/BiVO_4_ core/shell nanorods	1 m Na_2_SO_3_	5.3	^[^ ^58^ ^]^
		Sb: SnO_2_ nanotubes/BiVO_4_ shell	1 m Na_2_SO_3_	5.2	^[^ ^73^ ^]^
	1D/1D	SnO_2_ nanodendrite arrays/BiVO_4_ films	0.3 m Na_2_SO_4_	0.6	^[^ ^74^ ^]^
	1D/2D	Screw‐like SnO_2_ nanostructures/CdS QDs	0.25 m Na_2_S + 0.35 m Na_2_SO_3_	9.9 [0 V vs SCE]	^[^ ^41^ ^]^
	3D/0D	F: SnO_2_/TiO_2_/BiVO_4_ composite inverse opals	0.5 m Na_2_SO_4_	4.11	^[^ ^37^ ^]^
	3D/1D	Sb: SnO_2_ macropore/ Fe_2_O_3_ nanorods	1 m NaOH	1.1	^[^ ^75^ ^]^
		SnO_2_ nanobowl arrays/CdS nanorods	0.25 m Na_2_S + 0.35 m Na_2_SO_3_	3	^[^ ^76^ ^]^
		F: SnO_2_ inverse opals/CdS NRs/CdSe clusters	0.25 m Na_2_S + 0.35 m Na_2_SO_3_	9.2	^[^ ^77^ ^]^
	3D/2D	SnO_2_ microspheres/ nanosheets/TiO_2_/BiVO_4_	0.5 m Na_2_SO_4_ + 0.1 m Na_2_SO_3_	5.03	^[^ ^35^ ^]^
WO_3_	1D/0D	WO_3_/BiVO_4_ core/shell nanorods	1 m Na_2_SO_3_	4.15	^[^ ^78^ ^]^
		WO_3_/Fe_2_O_3_ core/shell needles	0.1 m phosphate	1.75	^[^ ^79^ ^]^
	2D/0D	WO_3_ nanosheets/barium bismuth niobate films	0.5 m Na_2_SO_4_	2.23	^[^ ^43^ ^]^
		WO_3_/BiVO_4_ core/shell nanosheets	0.5 m Na_2_SO_4_	1.62	^[^ ^80^ ^]^
	2D/1D	WO_3_ nanoplates/Bi_2_S_3_ nanorods	0.1 m Na_2_S + 0.1 m Na_2_SO_3_	10.2	^[^ ^81^ ^]^
		WO_3_ nanoplates/Fe_2_O_3_ nanorods	1 m NaOH	0.25	^[^ ^39^ ^]^
	3D/0D	WO_3_/BiVO_4_ composite inverse opals	0.5 m Na_2_SO_4_	0.8	^[^ ^40^ ^]^
		Brochosomes‐like WO_3_/BiVO_4_ arrays	0.5 m Na_2_SO_4_	3.13	^[^ ^36^ ^]^
ZnO	1D/0D	ZnO/Fe_2_O_3_ core/shell nanowires	1 m NaOH	1.3	^[^ ^82^ ^]^
		ZnO nanorods/BiVO_4_ nanoparticles	0.2 m Na_2_SO_4_	1.9	^[^ ^83^ ^]^
		N: ZnO/Mo‐doped BiVO_4_ bunched nanorods	0.5 m Na_2_SO_4_	3.62	^[^ ^84^ ^]^
		ZnO nanorods/CdS/ZnFe_2_O_4_ nanoparticles	0.1 m Na_2_S + 0.2 m Na_2_SO_3_	9.16 [0.4 V vs SCE]	^[^ ^42^ ^]^
		ZnO nanorods/CdSe layer	0.25 m Na_2_S + 0.35 m Na_2_SO_3_	11.5	^[^ ^85^ ^]^
		Al: ZnO nanowires/ZnFe_2_O_4_ shell	0.1 m Na_2_SO_4_	1.72	^[^ ^86^ ^]^
	1D/1D	ZnO nanodendrite arrays/BiVO_4_ films	0.3 m Na_2_SO_4_	2.45	^[^ ^68^ ^]^
		Branched ZnO nanowire arrays/CdS nanoparticles	0.5 m Na_2_S	3.58	^[^ ^87^ ^]^
	2D/1D	ZnO nanorod‐nanosheet/CdS quantum dots	0.5 m Na_2_SO_4_	4.25 [0.4 V vs Ag/AgCl]	^[^ ^88^ ^]^
	3D/0D	ZnO inverse opals/BiVO_4_ films	0.2 m Na_2_SO_4_	4.2	^[^ ^89^ ^]^
		Al: ZnO inverse opals/BiVO_4_ films	Phosphate buffer	1.5	^[^ ^90^ ^]^

**Figure 4:**
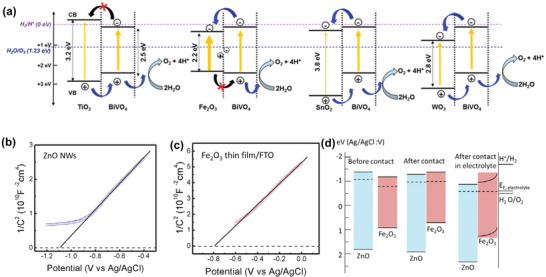
a) The band alignment of different heterojunction based on BiVO_4_ composite films. Reproduced with permission.^[^ ^104^ ^]^ Copyright 2016, Elsevier Ltd. b,c) Mott–Schottky plots of ZnO NWs and Fe_2_O_3_ thin film. d) Schematic of the band alignment between ZnO NWs and Fe_2_O_3_ thin film. Reproduced with permission.^[^ ^82^ ^]^ Copyright 2015, American Chemical Society.

In host/guest photoanodes, the contact between host and guest semiconductors favors the formation of type‐II heterojunctions due to the more positive band edges of host materials than that of guest materials. In type‐II heterojunctions, photogenerated electrons transfer from the conduction band (CB) of guest materials to the more conductive host materials and finally reach the counter electrode, while holes facilely move in the opposite direction, thus resulting in a spatial separation of electron–hole pairs and a depressed charge recombination.^[^ ^100^, ^101^, ^102^, ^103^ ^]^ Lee et al. investigated the construction of heterojunction films consisting of guest BiVO_4_ with a series of host materials, such as Fe_2_O_3_, TiO_2_, SnO_2_, and WO_3_.^[^ ^104^ ^]^ As shown in **Figure** **4**a, SnO_2_/BiVO_4_ and WO_3_/BiVO_4_ host/guest films can form a type‐II heterojunction due to the matched band alignment. Host/guest photoanodes showed an increased photocurrent at 1.23 V versus RHE compared to pristine BiVO_4_ films. However, the direct combination of Fe_2_O_3_/BiVO_4_ and TiO_2_/BiVO_4_ are not beneficial to the charge transfer in the heterojunction due to the unmatched band alignment, resulting in a decreased photocurrent. The band alignment between host materials and guest materials can be further judged with experimental techniques. The Mott–Schottky plots technique is frequently adopted to acquire the flat band potential, which is close to the CB edge.^[^ ^105^ ^]^ The VB edge can be calculated by the sum of the CB edge and the bandgap value. Hsu et al. measured the flat‐band potential of ZnO and Fe_2_O_3_ (Figure 4b,c).^[^ ^82^ ^]^ Before contact, the flat‐band potential of ZnO (−1.09 V vs Ag/AgCl) is more negative than that of Fe_2_O_3_ (−0.79 V vs Ag/AgCl). After contact, electrons in the Fermi level of ZnO migrate to Fe_2_O_3_ due to its more considerable work function. As the carrier concentration of Fe_2_O_3_ is lower than ZnO, the energy difference between CB and Fermi levels in ZnO is larger than that in Fe_2_O_3_. Thus, after the alignment of Fermi levels, the CB edge of ZnO becomes more positive than that of Fe_2_O_3_, achieving a ZnO/Fe_2_O_3_ heterojunction (Figure 4d). Bai et al. reported the application of WO_3_/Fe_2_O_3_ heterojunction as the host/guest photoanode.^[^ ^39^ ^]^ The flat band potentials of WO_3_ NSs and Fe_2_O_3_ NRs were measured as −0.33 and −0.42 V versus Ag/AgCl, respectively. The more positive CB edge of WO_3_ NSs than that of Fe_2_O_3_ NRs favors the formation of ZnO/Fe_2_O_3_ heterojunction. Other type‐II heterojunctions, such as WO_3_/barium bismuth niobate^[^ ^43^ ^]^ and SnO_2_/CdS^[^ ^76^ ^]^ have also been reported as host/guest photoanodes, which is a fundamental role in the promotion of charge separation.

## 4 Targeted Enhancement Strategies for Host/Guest Photoanodes

The emergence of host/guest architectures in photoanodes can enhance visible light absorption as well as the separation and collection of charge carriers, thus dramatically improving photocurrent and STH efficiency of photoanodes. Based on the construction feature of host/guest architectures in photoanodes, three targeted enhancement strategies including i) light trapping effect for enhanced light absorption; ii) optimization of conductive host scaffolds for improved charge separation; iii) hierarchical structure engineering for simultaneous enhancement of light absorption and charge separation were studied. This section aims to summarize and highlight recent state‐of‐the‐art progress in the architectural design of host/guest photoanodes with these enhancement strategies.

### 4.1 Light Trapping Effect

*η*_abs_ is determined by the light‐harvesting efficiency. Sufficient light‐harvesting is a prerequisite to enhancing PEC performance of host/guest photoanodes. The absorption of more incident photons generates more electron–hole pairs for the chemical reaction in a PEC cell. Light trapping induced by multiple light scattering or slow‐photon effect can prolong the optical path to increase the *η*_abs_. Concurrently, the short hole diffusion path can be well maintained to avoid the adverse impact on *η*_sep_.^[^ ^106^, ^107^, ^108^ ^]^ Light trapping effects are usually achieved in well‐designed porous or periodic host/guest nanostructured photoanodes.

**Figure 5:**
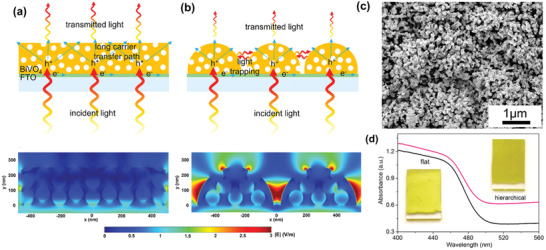
Schematic of light penetration and corresponding FDTD simulation of a) flat film and b) nanosphere arrays. c) SEM image of WO_3_/BiVO_4_ nanosphere arrays. d) UV–vis diffuse reflectance spectra. Reproduced with permission.^[^ ^66^ ^]^ Copyright 2017, American Chemical Society.

Multiple light scattering occurs at the void areas of nanostructures, effectively prolonging the optical path at a fixed film thickness, which enhances the light‐harvesting capability. Zhou et al. investigated the light‐matter interaction in BiVO_4_ flat films and nanosphere arrays with relative film thickness (**Figure** **5**).^[^ ^66^ ^]^ The incident light penetrates the film directly without scattering in the flat films, as reflected by the evenly distributed electric field (Figure 5a). When the distance between adjacent nanospheres approaches the wavelength of the incident light, the incident light is scattered and trapped in the nanosphere arrays, resulting in an intensified electric field in the void areas (Figure 5b) as observed through finite difference time domain (FDTD) simulation. WO_3_/BiVO_4_ nanosphere arrays with sufficient voids were confirmed by scanning electron microscope (SEM) (Figure 5c). The UV–vis diffuse reflectance spectra shows that WO_3_/BiVO_4_ nanosphere arrays could adsorb more incident photons than flat WO_3_/BiVO_4_ films (Figure 5d).

**Figure 6:**
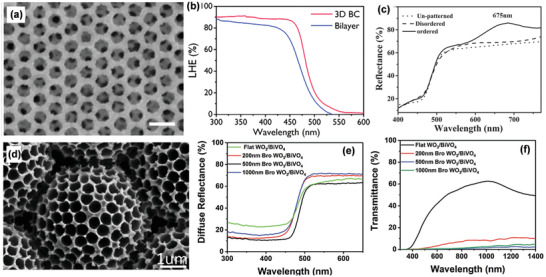
3D ZnO IOs/BiVO_4_ films: a) SEM image; b) Light‐harvesting efficiency. a,b) Reproduced with permission.^[^ ^89^ ^]^ Copyright 2018, American Chemical Society. WO_3_/BiVO_4_ core/shell IOs: c) Diffuse reflectance spectra. Reproduced with permission.^[^ ^40^ ^]^ Copyright 2017, Wiley‐VCH. Brochosomes‐like WO_3_/BiVO_4_ arrays: d) SEM image; e) Diffuse reflectance spectra; f) Transmittance spectra. d,e) Reproduced under the terms of the Creative Commons CC‐BY license.^[^ ^35^ ^]^ Copyright 2019, The Authors. Published by Wiley‐VCH.

3D IOs are periodic porous structures that can provide plenty of controllable void areas, which enhances the light trapping capability.^[^ ^109^, ^110^ ^]^ 3D host IOs are usually fabricated by the template‐assisted method. In a typical synthesis process, the precursors of the host semiconductor are first infiltrated into self‐assembled 3D periodic opal templates. 3D host IOs can be obtained through the removal of the opals template by a postannealing or chemical corrosion. The photoactive guest semiconductors are then coated onto the 3D host IOs by spin‐coating or electrodeposition technique to create the host/guest photoanodes with periodic porous structures.^[^ ^44^ ^]^ Kim et al. reported the coating of guest BiVO_4_ thin layer on host 3D ZnO IOs to form typical periodic porous structures for improved PEC application.^[^ ^89^ ^]^ As shown in **Figure** **6**a, the periodic and porous structure of ZnO IOs is well retained even after the coating of BiVO_4_ films. 3D ZnO IOs/BiVO_4_ films exhibited a 50% higher light‐harvesting efficiency than ZnO/BiVO_4_ bilayer films in the wavelength range of 450−500 nm (Figure 6b). The enhanced light absorption can be ascribed to the multiple light‐scattering effects within the IOs. Such multiple light scattering can also be achieved in other host/guest IOs, such as SnO_2_ IOs/BiVO_4_,^[^ ^111^ ^]^ and ZnO IOs/Zn*_x_* Cd_1−_ *_x_* Se.^[^ ^112^ ^]^

3D IOs can also achieve an additional slow photon effect, further contributing to the light trapping capability.^[^ ^113^ ^]^ Due to the periodic variation of the refractive index, 3D IOs as a photonic crystal can present an apparent photonic stopband. The wavelength of incident light within the photonic stopband cannot transmit through the 3D IOs, forming a sharp reflection peak. At the edges of the photonic stopband, photons propagating through the material exhibit a strongly reduced group velocity, called slow photons, which can significantly increase the probability of absorption.^[^ ^114^ ^]^ Zhang et al. investigated the photonic stopband of WO_3_/BiVO_4_ core/shell IOs.^[^ ^40^ ^]^ When compared to disordered WO_3_/BiVO_4_/Co‐Pi, and unpatterned WO_3_/BiVO_4_/Co‐Pi, only ordered WO_3_ IOs/BiVO_4_/Co‐Pi presented a Bragg reflection peak at 675 nm due to the stopband reflection effect (Figure 6c). The slow photon effect could thus enhance the light‐harvesting efficiency of ordered WO_3_ IOs/BiVO_4_/Co‐Pi near the stopband edge.

Apart from 3D IOs, other periodic porous host scaffolds can also be used for light trapping. Zhang et al. reported 3D brochosomes like WO_3_ arrays as the host scaffolds (Figure 6d) for guest BiVO_4_ films.^[^ ^36^ ^]^ A hollow spherical core with plenty of periodic pits makes up one brochosomes like unit, and these units self‐assemble to form the final brochosomes like architectures. Such ordered, hollow and porous structures not only provide a high specific surface area but also induce multiple light scattering effects. Hence, brochosomes like WO_3_ arrays/BiVO_4_ films show a lower reflectance intensity in the range of 300−500 nm (Figure 6e) and lower transmittance (Figure 6f) in a broadband wavelength than flat WO_3_/BiVO_4_ films. This suggests that brochosomes‐like architectures have superior light‐harvesting abilities.

Therefore, the multiple light scattering and slow photon effect in the periodic and porous architectures are beneficial to light trapping, resulting in an improvement in the light‐harvesting efficiency and *η*_abs_. The improved light‐harvesting efficiency of host/guest photoanodes can be verified by diffuse reflectance spectra and FDTD simulations.

### 4.2 Optimization of Conductive Host Scaffolds

**Figure 7:**
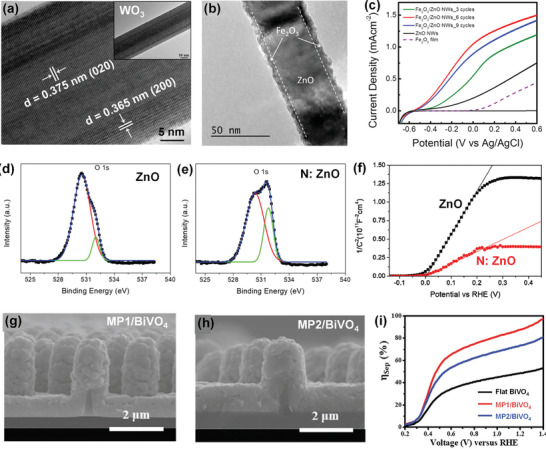
a) HRTEM image of WO_3_ nanoneedles. Reproduced with permission.^[^ ^79^ ^]^ Copyright 2014, Elsevier Ltd. ZnO/Fe_2_O_3_ core/shell NWs: b) TEM image; c) LSV plots. b,c) Reproduced with permission.^[^ ^82^ ^]^ Copyright 2015, American Chemical Society. d–f) XPS spectra and Mott‐Schottky plots of ZnO and N: ZnO NRs. d‐f) Reproduced with permission.^[^ ^84^ ^]^ Copyright 2018, Royal Society of Chemistry. F: SnO_2_ micropillar nanostructures/BiVO_4_: g–h): SEM images of MP1/BiVO_4_ and MP2/BiVO_4_; i) *η*_sep_ at different potentials. g‐h) Reproduced with permission.^[^ ^125^ ^]^ Copyright 2021, Wiley‐VCH.

Host scaffolds with high electron‐extraction ability can effectively accelerate the electron transfer from guest photoactive semiconductors to the conductive substrates, reducing the bulk recombination of photogenerated electron−hole pairs.^[^ ^115^, ^116^, ^117^ ^]^ 1D nanostructures with a reduced quantity of dead ends and fewer grain boundaries could provide direct and ordered channels for charge transport.^[^ ^118^, ^119^, ^120^ ^]^ Thus, various 1D host NRs or NWs have been reported as scaffolds. Tao et al. reported the high‐aspect‐ratio WO_3_ nanoneedles, fabricated with a seed‐mediated hydrothermal reaction method, as the host scaffolds for Fe_2_O_3_ NPs.^[^ ^79^ ^]^ The single crystalline structure with (020) orientation of monoclinic WO_3_ can be observed by the high‐resolution transmission electron microscopy  (HRTEM), as shown in **Figure** **7**a. Due to its single crystallinity, monoclinic WO_3_ has inherently remarkable electron transport properties that greatly inhibited electron−hole recombination in Fe_2_O_3_ NPs. Yu‐Kuei et al. reported 1D ZnO/Fe_2_O_3_ core/shell nanostructures as the host/guest photoanode.^[^ ^82^ ^]^ Fe_2_O_3_ films with a thickness of a few nanometers were coated on 1D ZnO NWs that has a thickness of 80 nm (Figure 7b). By optimizing Fe_2_O_3_ films thickness, 1D ZnO/Fe_2_O_3_ photoanode achieved a photocurrent density of 1.5 mA cm^−2^ at 0.6 V versus AgCl, while Fe_2_O_3_ films only exhibited a photocurrent density of 0.5 mA cm^−2^ (Figure 7c).

Element doping is an effective strategy to improve the electrical conductivity of host scaffolds, which can further enhance its charge collection ability.^[^ ^121^, ^122^, ^123^ ^]^ The more efficient collection of the photogenerated carriers in the photoanodes can be indicated by the higher carrier density. Kim et al. reported nitrogen‐doped ZnO (N: ZnO) NRs as the host scaffold.^[^ ^84^ ^]^ Figure 7d,e shows the O 1s X‐ray photoelectron spectroscopy (XPS) spectra of ZnO NRs and N: ZnO NRs, respectively. After introducing N dopants, the oxygen vacancy peak at 531.8 eV is highly intensified, indicating an increase in the oxygen vacancies in N: ZnO NRs. Oxygen vacancies could enhance electron densities, which can be experimentally calculated from the slope of Mott–Schottky plots. The Mott–Schottky plots of ZnO NRs and ZnO: N NRs are shown in Figure 7f. The charge carrier density of ZnO: N NRs (8.58 × 10^18^ cm^−3^) is threefold that of undoped ZnO NRs (2.42 × 10^18^ cm^−3^), suggesting an improvement in the charge collection in ZnO: N NRs. Similarly, 1D Sb: SnO_2_ nanotubes,^[^ ^73^ ^]^ and Sn: In_2_O_3_ NWs^[^ ^124^ ^]^ were reported as conductive host scaffolds to improve charge collection of the guest films. Sucheol et al. further investigated the direct contribution of conductive scaffolds on *η*_sep_.^[^ ^125^ ^]^ The patterned F: SnO_2_ micropillar nanostructures were developed using printing and spray pyrolysis techniques. By adjusting the periods, two types of F: SnO_2_ micropillar nanostructures were obtained, denoted as MP1 and MP2. MP1 and MP2 have the same height of 1.9 µm and diameter of 500 nm, but different periods of 2 and 4 µm, respectively (Figure 7h,g). The *η*_sep_ of both MP1/BiVO_4_ (88.8%) and MP2/BiVO_4_ (74.2%) host/guest photoanodes at 1.23 V versus RHE are higher than that of flat BiVO_4_ films (49.2%) (Figure 7i), suggesting that conductive scaffolds can significantly improve *η*_sep_.

**Figure 8:**
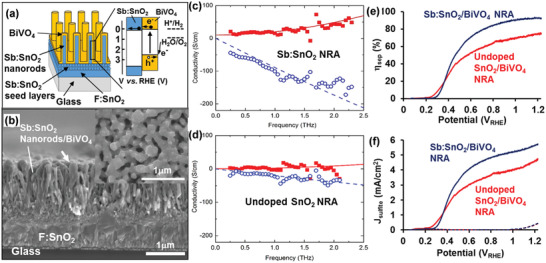
a) Schematic of the structure and energy band diagram of Sb: SnO_2_/BiVO_4_ NRA on FTO substrate. b) The cross‐section and inset top‐view SEM images of Sb: SnO_2_/BiVO_4_ NRA thin films. THz‐TDS spectra of c) Sb: SnO_2_ NRA and d) undoped SnO_2_ NRA. e,f) LSV plots and *η*_sep_ of Sb: SnO_2_/BiVO_4_ NRA thin films and undoped SnO_2_/BiVO_4_ NRA thin films with sacrificial reagents. Reproduced with permission.^[^ ^58^ ^]^ Copyright 2016, American Chemical Society.

More techniques have been applied to investigate the influence of conductive scaffolds. The terahertz time‐domain spectroscopy (THz‐TDS) spectra is a contact‐free technique to investigate the carrier dynamics over nanometer length scales and the intrinsic conductivity within individual host scaffolds.^[^ ^126^, ^127^ ^]^ Zhou et al. prepared thin layers of guest BiVO_4_ coated onto host Sb: SnO_2_ nanorod arrays (NRA) (**Figure** **8**a,b).^[^ ^58^ ^]^ As shown in the THz conductivity spectra (Figure 8c,d), the electrical conductivity values of Sb: SnO_2_ and undoped SnO_2_ NRA were calculated as 33.2 ± 6.4 S cm^−1^ and 7.4 ± 1.8 S cm^−1^, respectively. The greater electrical conductivity of Sb: SnO_2_ NRA scaffold enhanced *η*_sep_ from 58% to 71% at 0.6 V versus RHE for Sb: SnO_2_/BiVO_4_ host/guest photoanodes under backside illumination (Figure 8e). This resulted in a higher photocurrent performance, compared to undoped SnO_2_/BiVO_4_ NRA host/guest photoanodes (Figure 8f).

**Figure 9:**
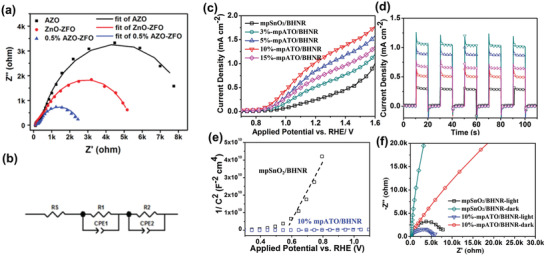
Al: ZnO NWs/ZnFe_2_O_4_ films: a,b) Nyquist EIS plots and the equivalent circuit. a,b) Reproduced with permission.^[^ ^86^ ^]^ Copyright 2016, Royal Society of Chemistry. Sb: SnO_2_ macropores/Fe_2_O_3_ NRs: c) LSV plots; d) LSV plots under chopped illumination; e) Mott–Schottky plots; f) Nyquist EIS plots. c‐f) Reproduced under the terms of the Creative Commons CC‐BY license.^[^ ^75^ ^]^ Copyright 2015, The Authors. Published by Wiley‐VCH.

The charge transport capability of the host scaffolds in host/guest photoanodes is also frequently investigated with the photoassisted electrical impedance spectroscopy (EIS), which can reveal the charge transfer at the photoanode surface and in the bulk semiconductor.^[^ ^128^ ^]^ Nyquist plots from the EIS test can be fitted with two resistance and capacitance (RC) circuit models. In the equivalent circuit, the series resistance (*R*_s_), first RC circuit (R_1_ and CPE_1_), and charge transfer resistance (R_2_) can be acquired. R_1_ and CPE_1_ are determined by the charge transport process, and R_2_ is the charge transfer resistance across the semiconductor‐liquid junction interface, which corresponds to the biggest arc at the lower frequency.^[^ ^63^, ^129^ ^]^ Xu et al. reported the in situ growth of photoactive guest ZnFe_2_O_4_ on host Al: ZnO NWs as the host/guest photoanode.^[^ ^86^ ^]^ Al: ZnO NWs/ZnFe_2_O_4_ films showed a lower R_1_ (158.5 Ω) than pristine ZnO NWs/ZnFe_2_O_4_ (309.4 Ω), indicating a higher conductivity of Al: ZnO scaffolds for the transport of photogenerated electrons (**Figure** **9**a,b). Zhang et al. reported Sn: In_2_O_3_ NWs/BiVO_4_ films as the host/guest photoanode. The Sn: In_2_O_3_ NWs with an average diameter of 110 nm and a length of more than 10 µm were grown on a FTO substrate, providing a direct conductive pathway for electron transport. The reduced *R*_s_ of Sn: In_2_O_3_ NWs/BiVO_4_/rGO (2.5 Ω) by EIS measurements could be partly attributed to the conductive Sn: In_2_O_3_ NWs, compared to pristine In_2_O_3_ NWs/BiVO_4_ (243.3 Ω).

Apart from 1D conductive host scaffolds, 3D conductive host scaffolds have also been reported for improved charge collection. Zhang et al. prepared 3D Al‐doped ZnO IOs to be used as efficient electron collectors for BiVO_4_ films, and it showed enhanced light‐harvesting and electron transport from BiVO_4_ to Al: ZnO IOs.^[^ ^90^ ^]^ The optimization of dopant concentration in host metal oxides is also a critical factor for efficient PEC performance of host/guest photoanodes. Xu et al. investigated the effect of dopant concentration of Sb element in SnO_2_ macropores as the host scaffolds (Figure 9c,d).^[^ ^75^ ^]^ The maximum photocurrent was achieved with 10% of dopant concentration. Further increase of dopant concentration to 15% resulted in a decline in the photocurrent due to the detrimental electron scattering effect. Mott–Schottky plots and EIS were further employed to verify the contribution of optimized scaffolds on charge separation. The introduced conductive host scaffolds may further act as the doping source for guest photoactive materials, resulting in significant carrier density improvement of both host and guest semiconductors due to the synergic doping effects (Figure 9e,f). For example, Zhang et al. reported Sn: In_2_O_3_ NWs/Fe_2_O_3_ host/guest photoanode for enhanced PEC performance.^[^ ^124^ ^]^ In the postannealing treatment, Sn atoms diffuse from Sn: In_2_O_3_ NWs to the Fe_2_O_3_ nanostructures. The doping of the guest semiconductor resulted in efficient electron transfer from the guest Sn‐doped Fe_2_O_3_ to the Sn: In_2_O_3_ electron collector, which enhanced the PEC performance.

Therefore, the charge separation efficiency can be promoted by having conductive host scaffolds in host/guest photoanodes. The impact of conductive platforms can be investigated experimentally with the Mott–Schottky plots, THz‐TDS spectra, and photoassisted EIS.

### 4.3 Hierarchical Structure Engineering

Hierarchical nanostructures are constructed by the secondary growth of nanostructures on preacquired nanostructures, which would inherently increase the surface areas for mass transfer.^[^ ^130^, ^131^ ^]^ Through hierarchical structure engineering, several enhancement strategies can be combined within one host/guest photoanode, making it possible to improve *η*_abs_ and *η*_sep_ simultaneously. Typical fabrication techniques for hierarchical host/guest nanostructures are summarized as follows: i) direct growth of 1D guest nanostructures on 1D, 2D, or 3D host scaffolds; ii) coating of guest films on the fabricated hierarchical host scaffolds.

**Figure 10:**
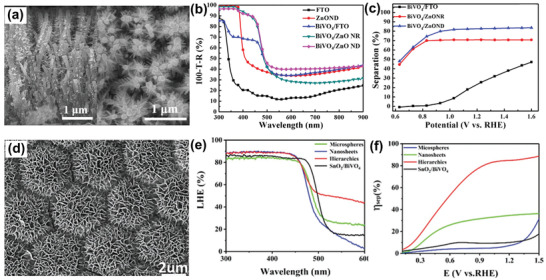
Hierarchical ZnO nanodendrite arrays/BiVO_4_ films: a) The cross‐section and inset top‐view SEM images of ZnO nanodendrite arrays; b) Light‐harvesting efficiency at different wavelengths; c) *η*_sep_. a‐c) Reproduced with permission.^[^ ^68^ ^]^ Copyright 2017, Elsevier Ltd. Hierarchical ternary SnO_2_/TiO_2_/BiVO_4_ arrays: d) SEM image of hierarchical SnO_2_ arrays; e) Light‐harvesting efficiency; f) *η*_sep_. d‐f) Reproduced under the terms of the Creative Commons CC‐BY license.^[^ ^35^ ^]^ Copyright 2020, The Authors. Published by Wiley‐VCH.

Compared to single‐dimensional host/guest nanostructures, hierarchical host/guest nanostructures inherently exhibit an enlarged surface area and volumes of the depletion regions, resulting in more efficient charge separation or light absorption in host/guest photoanodes. Yang et al. investigated the contribution of 1D/1D ZnO nanodendrite arrays to the PEC performance of guest BiVO_4_ thin films.^[^ ^68^ ^]^ Hierarchical ZnO nanodendrite host scaffolds were fabricated by the secondary growth of 1D ZnO branches on 1D ZnO NRs (**Figure** **10**a,b). As shown in Figure 10b, both ZnO NRs/BiVO_4_ and hierarchical ZnO nanodendrite arrays/BiVO_4_ exhibit more significant light‐harvesting efficiency than flat BiVO_4_ films. Additionally, hierarchical ZnO nanodendrite arrays/BiVO_4_ films exhibit higher *η*_sep_ than ZnO NRs/BiVO_4_ and flat BiVO_4_ films (Figure 10c), suggesting the extraordinary promotion effect of hierarchical nanostructures on charge separation. Similarly, Zhang et al. reported screw‐like SnO_2_ nanostructures through the growth of 2D SnO_2_ NSs onto 1D single‐crystalline SnO_2_ NRs. The resultant hierarchical 1D/2D host scaffolds offer a large surface‐to‐volume ratio for mass transfer.^[^ ^41^ ^]^ Through adjusting the coverage and the distance between adjacent SnO_2_ NSs, a significant increase in light absorption by ≈33% is achieved for 1D/2D SnO_2_ NRs/SnO_2_ NSs as compared to 1D SnO_2_ NRs. Liu et al. reported the 2D/1D ZnO NSs/NRs mixed dimensional architectures as the host scaffolds.^[^ ^88^ ^]^ The light absorption in the 1D/1D branched ZnO NRs, and 2D/1D ZnO NSs/NRs nanoarchitecture was investigated by FDTD simulation. |E| distributions in 2D/1D ZnO NSs/NRs are more efficient and uniform than that of 1D/1D branched ZnO NRs, indicating an effective penetration of light to the bottom areas. Furthermore, Pan et al. reported the simultaneous enhancement of *η*_abs_ and *η*_sep_ in 3D hierarchical ternary SnO_2_/TiO_2_/BiVO_4_ arrays.^[^ ^35^ ^]^ In such composite photoanodes, 3D SnO_2_ hollow microsphere arrays coated with 2D SnO_2_ NSs act as the hierarchical host scaffolds (Figure 10d). TiO_2_ and BiVO_4_ take the roles of a hole blocking layer and a visible absorption layer, respectively. Due to a large specific surface area and efficient interface contact, hierarchical SnO_2_/TiO_2_/BiVO_4_ arrays achieved a higher light‐harvesting efficiency (Figure 10e) and *η*_sep_ (Figure 10f) than SnO_2_ NSs/TiO_2_/BiVO_4_ and SnO_2_ NSs microsphere/TiO_2_/BiVO_4_.

**Figure 11:**
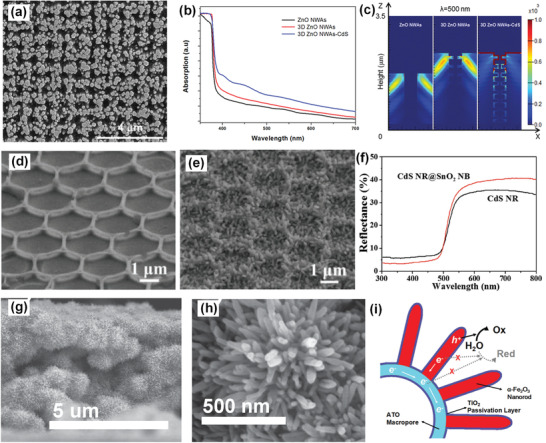
1D/1D periodic branched ZnO NWs/CdS: a) SEM image of branched ZnO NWs; b) UV–vis absorption spectra; c) Simulated optical absorption. a‐c) Reproduced with permission.^[^ ^87^ ^]^ Copyright 2016, Wiley‐VCH. 2D/1D periodic SnO_2_ nanobowls/CdS NRs: d) SEM image of periodic SnO_2_ nanobowls; e) SEM image of periodic SnO_2_ nanobowls/CdS NRs; f) UV–vis diffuse reflectance spectra. d‐f) Reproduced with permission.^[^ ^76^ ^]^ Copyright 2018, Wiley‐VCH. 3D/1D periodic SnO_2_ macropores/Fe_2_O_3_ NRs: g,h) SEM images of SnO_2_ macropores/Fe_2_O_3_ NRs; i) The sketch of charge transport and separation. g‐i) Reproduced under the terms of the Creative Commons CC‐BY license.^[^ ^75^ ^]^ Copyright 2015, The Authors. Published by Wiley‐VCH.

The light‐trapping effect can also be achieved in periodic hierarchical nanostructures. Bai et al. reported the photoresist template‐assisted growth of periodic 1D/1D branched ZnO NWs as the hierarchical host scaffolds (**Figure** **11**a).^[^ ^87^ ^]^ Due to an increased roughness factor, 1D/1D branched ZnO NWs show a higher UV light absorption than 1D ZnO NWs (Figure 11b). The light‐trapping effect was further investigated by the FDTD simulation. 1D/1D branched ZnO NWs show larger electric intensity than 1D ZnO NWs (Figure 11c). After coating with CdS films, visible light trapping effect is achieved as reflected by the red region. Wang reported the growth of 1D guest CdS NRs on 3D periodic SnO_2_ nanobowls as the hierarchical host/guest photoanode.^[^ ^76^ ^]^ The periodic structure of SnO_2_ nanobowls is not disrupted after the growth of CdS NRs (Figure 11d,e), which is essential to the formation of multiple light scattering. SnO_2_ nanobowls/CdS NRs show a lower reflectance between 300 and 500 nm but a considerably higher reflectance above 500 nm (Figure 11f). As the absorption edge of CdS is 540 nm, the higher reflectance above 500 nm can be ascribed to the multiple light scattering effect. The lower reflectance between 300 and 500 nm indicates more efficient light absorption and lower surface reflection. The light‐trapping effect can be complemented with multiple scattering by constructing hierarchical 3D IOs/1D NRs architectures. Xu et al. reported 3D Sb: SnO_2_ macropores/1D Fe_2_O_3_ NRs as the hierarchical host/guest photoanodes.^[^ ^75^ ^]^ 1D Fe_2_O_3_ NRs were grown on the walls of Sb: SnO_2_ macropores (Figure 11g,h). The sketch of charge transport and separation is shown in Figure 11i. Sb: SnO_2_ macropores take the role of extracting electrons from Fe_2_O_3_ NRs. The periodic and porous structure of Sb: SnO_2_ macropores is also beneficial to light‐harvesting. Similarly, 1D CdS NRs have also been grown on 3D F: SnO_2_ IOs for the simultaneous improvement of *η*_abs_ and *η*_sep_.^[^ ^77^ ^]^

Different enhancement strategies, such as increased specific surface areas, light trapping effect, and introduction of conductive scaffolds, can be combined into one hierarchical host/guest photoanode. Due to the synergic effects, hierarchical host/guest photoanodes usually exhibit an improved PEC performance compared to the corresponding pristine host/guest photoanodes.

## 5 Challenges and Perspectives

Owing to the significant enhancements of light‐harvesting as well as the separation and collection of charge carriers, construction of host/guest photoanodes is one of the most effective strategies to improve the PEC performance dramatically. The recent state‐of‐the‐art progress in the architectural design of host/guest photoanodes with integrated enhancement strategies including i) light trapping effect; ii) optimization of conductive host scaffolds; and iii) hierarchical structure engineering are summarized and highlighted.

However, photoactive guest materials may still suffer from slow OER kinetics, and the mere introduction of host scaffolds cannot overcome this problem. Surface modification of photoanodes with active OER catalysts such as Co‐Pi NPs^[^ ^83^ ^]^ and Ni‐Fe‐OH NSs,^[^ ^132^ ^]^ is one of the common strategies to accelerate OER kinetics and reduce surface trap states. The detailed contribution of OER catalysts in PEC application can be found in many other reviews, which is not summarized in the scope of this review. Another challenge is in obtaining the uniform coating of photoactive thin films on the host scaffolds, especially for the hierarchical host scaffolds with a high aspect ratio. Traditional coating techniques such as spin‐coating, dip‐coating, and chemical vapor deposition may not fully infiltrate precursors into the bottom areas of the host scaffolds. Pressure tuned atomic layer deposition (ALD) has been reported for the uniform coating of photoactive films on porous structures.^[^ ^133^ ^]^ However, only certain photoactive semiconductors, such as TiO_2_ and Fe_2_O_3_,^[^ ^115^, ^134^ ^]^ can be prepared by the ALD technique. Besides, the precursors for ALD applications are usually expensive.^[^ ^135^, ^136^, ^137^ ^]^ Thus, it is necessary to explore other methods for the uniform coating of photoactive thin films on the host scaffolds at a low cost.

Host/guest architectures are not only applicable for PEC water splitting, but also for electrocatalyst,^[^ ^138^ ^]^ solar cell,^[^ ^139^ ^]^ and ion battery.^[^ ^140^, ^141^ ^]^ Based on the review of various nanostructured host scaffolds, an ideal host scaffold should fulfill the following requirements: low cost, high chemical stability, superior electrical conductivity, and periodic architectures. With the help of host scaffolds, the optical and electrical properties of various guest materials can be improved simply in constructing host/guest structures. However, to date, none of the reported host scaffolds can be scaled up for practical and commercial applications yet. Compared to ZnO, WO_3_, and pristine SnO_2_, F: SnO_2_ shows greatest potential as the candidate material for ideal host scaffolds. Although patterned F: SnO_2_ micropillar nanostructures and periodic F: SnO_2_ IOs have been reported as the host scaffolds, they still suffer from high cost and complicated fabrication processes. Research into alternative patterned F: SnO_2_ host scaffolds, with potential for mass production to replace flat F: SnO_2_ glass substrates, could generate substantial technological advances and commercial value in various fields. Moreover, the designing of fully integrated/wireless and partially integrated/wired PEC devices with host/guest structures should be developed for practical application.

## Conflict of Interest

The authors declare no conflict of interest.

## References

[1] F. Niu , D. Wang , F. Li , Y. Liu , S. Shen , T. J. Meyer , Adv. Energy Mater. 2019, 10, 1900399.

[2] W. Yang , R. R. Prabhakar , J. Tan , S. D. Tilley , J. Moon , Chem. Soc. Rev. 2019, 48, 4979. 31483417 10.1039/c8cs00997j

[3] H. He , A. Liao , W. Guo , W. Luo , Y. Zhou , Z. Zou , Nano Today 2019, 28, 100763.

[4] W. Tu , W. Guo , J. Hu , H. He , H. Li , Z. Li , W. Luo , Y. Zhou , Z. Zou , Mater. Today 2020, 33, 75.

[5] M. Faraji , M. Yousefi , S. Yousefzadeh , M. Zirak , N. Naseri , T. H. Jeon , W. Choi , A. Z. Moshfegh , Energy Environ. Sci. 2019, 12, 59.

[6] P. Zhang , X. F. Lu , D. Luan , X. W. Lou , Angew. Chem., Int. Ed. 2020, 132, 8205.

[7] H. Zhang , D. Li , W. J. Byun , X. Wang , T. J. Shin , H. Y. Jeong , H. Han , C. Li , J. S. Lee , Nat. Commun. 2020, 11, 4622. 32934221 10.1038/s41467-020-18484-8 PMC7493915

[8] G. Liu , W. S. Wong , M. Kraft , J. W. Ager , D. Vollmer , R. Xu , Chem. Soc. Rev. 2021, 50, 10674. 34369513 10.1039/d1cs00258a

[9] W. Wang , M. Xu , X. Xu , W. Zhou , Z. Shao , Angew. Chem., Int. Ed. 2020, 59, 136. 10.1002/anie.201900292 30790407

[10] S. E. Hosseini , M. A. Wahid , Int. J. Energy Res. 2020, 44, 4110.

[11] T. T. Lucas , M. A. Melo , A. L. Freitas , F. L. Souza , R. V. Gonçalves , Sol. Energy Mater. Sol. Cells 2020, 208, 110428.

[12] Y. Chen , X. Feng , Y. Liu , X. Guan , C. Burda , L. Guo , ACS Energy Lett. 2020, 5, 844.

[13] A. Landman , R. Halabi , P. Dias , H. Dotan , A. Mehlmann , G. E. Shter , M. Halabi , O. Naseraldeen , A. Mendes , G. S. Grader , Joule 2020, 4, 448.

[14] R. Wang , L. Wang , Y. Zhou , Z. Zou , Appl. Catal., B 2019, 255, 117738.

[15] A. Fujishima , K. Honda , Nature 1972, 238, 37. 12635268 10.1038/238037a0

[16] C. Ding , J. Shi , Z. Wang , C. Li , ACS Catal. 2016, 7, 675.

[17] N. Karjule , J. Barrio , L. Xing , M. Volokh , M. Shalom , Nano Lett. 2020, 20, 4618. 32407122 10.1021/acs.nanolett.0c01484

[18] H. Zhu , M. Zhao , J. Zhou , W. Li , H. Wang , Z. Xu , L. Lu , L. Pei , Z. Shi , S. Yan , Appl. Catal., B 2018, 234, 100.

[19] J. Joy , J. Mathew , S. C. George , Int. J. Hydrogen Energy 2018, 43, 4804.

[20] R. Tang , S. Zhou , Z. Zhang , R. Zheng , J. Huang , Adv. Mater. 2021, 33, 2005389. 10.1002/adma.202005389 33733537

[21] G. Zheng , J. Wang , H. Liu , V. Murugadoss , G. Zu , H. Che , C. Lai , H. Li , T. Ding , Q. Gao , Nanoscale 2019, 11, 18968. 31361294 10.1039/c9nr03474a

[22] F. Ning , M. Shao , S. Xu , Y. Fu , R. Zhang , M. Wei , D. G. Evans , X. Duan , Energy Environ. Sci. 2016, 9, 2633.

[23] R. Wang , X. Li , L. Wang , X. Zhao , G. Yang , A. Li , C. Wu , Q. Shen , Y. Zhou , Z. Zou , Nanoscale 2018, 10, 19621. 30325386 10.1039/c8nr06880a

[24] S. Chu , S. Vanka , Y. Wang , J. Gim , Y. Wang , Y.‐H. Ra , R. Hovden , H. Guo , I. Shih , Z. Mi , ACS Energy Lett. 2018, 3, 307.

[25] M. Zhou , Z. Liu , Q. Song , X. Li , B. Chen , Z. Liu , Appl. Catal., B 2019, 244, 188.

[26] G. Liu , Z. Li , T. Hasan , X. Chen , W. Zheng , W. Feng , D. Jia , Y. Zhou , P. Hu , J. Mater. Chem. A 2017, 5, 1989.

[27] X. L. Zheng , J. P. Song , T. Ling , Z. P. Hu , P. F. Yin , K. Davey , X. W. Du , S. Z. Qiao , Adv. Mater. 2016, 28, 4935. 27038367 10.1002/adma.201600437

[28] H. Zhu , Y. Zhang , J. Zhu , Y. Li , S. Jiang , N. Wu , Y. Wei , J. Zhou , Y. Song , J. Mater. Chem. A 2020, 8, 22929.

[29] J. B. Pan , S. Shen , L. Chen , C. T. Au , S. F. Yin , Adv. Funct. Mater. 2021, 2104269.

[30] J. Zheng , H. Zhou , Y. Zou , R. Wang , Y. Lyu , S. Wang , Energy Environ. Sci. 2019, 12, 2345.

[31] W. Yang , J. Moon , ChemSusChem 2019, 12, 1889. 30102017 10.1002/cssc.201801554

[32] W. Tian , C. Chen , L. Meng , W. Xu , F. Cao , L. Li , Adv. Energy Mater. 2020, 10, 1903951.

[33] H. Zheng , Y. Lu , K.‐H. Ye , J. Hu , S. Liu , J. Yan , Y. Ye , Y. Guo , Z. Lin , J. Cheng , Nat. Commun. 2021, 12, 91. 33398029 10.1038/s41467-020-20341-7 PMC7782821

[34] J. W. Yang , I. J. Park , S. A. Lee , M. G. Lee , T. H. Lee , H. Park , C. Kim , J. Park , J. Moon , J. Y. Kim , Appl. Catal., B 2021, 293, 120217.

[35] Q. Pan , A. Li , Y. Zhang , Y. Yang , C. Cheng , Adv. Sci. 2020, 7, 1902235. 10.1002/advs.201902235 PMC7001624 32042560

[36] Q. Pan , H. Zhang , Y. Yang , C. Cheng , Small 2019, 15, 1900924. 10.1002/smll.201900924 31165562

[37] H. Zhang , C. Cheng , ACS Energy Lett. 2017, 2, 813.

[38] K. Sivula , F. L. Formal , M. Gratzel , Chem. Mater. 2009, 21, 2862.

[39] S. Bai , X. Yang , C. Liu , X. Xiang , R. Luo , J. He , A. Chen , ACS Sustainable Chem. Eng. 2018, 6, 12906.

[40] H. Zhang , W. Zhou , Y. Yang , C. Cheng , Small 2017, 13, 1603840. 10.1002/smll.201603840 28165199

[41] Z. Zhang , C. Gao , Z. Wu , W. Han , Y. Wang , W. Fu , X. Li , E. Xie , Nano Energy 2016, 19, 318.

[42] S. Cao , X. Yan , Z. Kang , Q. Liang , X. Liao , Y. Zhang , Nano Energy 2016, 24, 25.

[43] B. Weng , C. R. Grice , J. Ge , T. Poudel , X. Deng , Y. Yan , Adv. Energy Mater. 2018, 8, 1701655.

[44] Z. Wang , X. Li , H. Ling , C. K. Tan , L. P. Yeo , A. C. Grimsdale , A. I. Y. Tok , Small 2018, 14, 1800395. 10.1002/smll.201800395 29665266

[45] B. Lamm , L. Zhou , P. Rao , M. Stefik , ChemSusChem 2019, 9, 12. 10.1002/cssc.201802566 30571851

[46] G. Liu , Y. Sheng , J. W. Ager , M. Kraft , R. Xu , EnergyChem 2019, 1, 100014.

[47] S.‐S. Yi , X.‐B. Zhang , B.‐R. Wulan , J.‐M. Yan , Q. Jiang , Energy Environ. Sci. 2018, 11, 3128.

[48] S. Kment , F. Riboni , S. Pausova , L. Wang , L. Wang , H. Han , Z. Hubicka , J. Krysa , P. Schmuki , R. Zboril , Chem. Soc. Rev. 2017, 46, 3716. 28397882 10.1039/c6cs00015k

[49] M. Xiao , B. Luo , Z. Wang , S. Wang , L. Wang , Sol. RRL 2020, 4, 1900509.

[50] D. Bae , B. Seger , P. C. Vesborg , O. Hansen , I. Chorkendorff , Chem. Soc. Rev. 2017, 46, 1933. 28246670 10.1039/c6cs00918b

[51] K. Takanabe , ACS Catal. 2017, 7, 8006.

[52] C. Jiang , S. J. A. Moniz , A. Wang , T. Zhang , J. Tang , Chem. Soc. Rev. 2017, 46, 4645. 28644493 10.1039/c6cs00306k

[53] X. Li , J. Yu , J. Low , Y. Fang , J. Xiao , X. Chen , J. Mater. Chem. A 2015, 3, 2485.

[54] S. Wang , T. He , P. Chen , A. Du , K. Ostrikov , W. Huang , L. Wang , Adv. Mater. 2020, 32, 2001385. 10.1002/adma.202001385 32406092

[55] Y. M. Choi , B. W. Lee , M. S. Jung , H. S. Han , S. H. Kim , K. Chen , D. H. Kim , T. F. Heinz , S. Fan , J. Lee , Adv. Energy Mater. 2020, 10, 2000570.

[56] B. He , S. Jia , M. Zhao , Y. Wang , T. Chen , S. Zhao , Z. Li , Z. Lin , Y. Zhao , X. Liu , Adv. Mater. 2021, 33, 2004406. 10.1002/adma.202004406 33734506

[57] D. A. Reddy , K. A. J. Reddy , M. Gopannagari , D. P. Kumar , T. K. Kim , Appl. Catal., B 2020, 269, 118761.

[58] L. Zhou , C. Zhao , B. Giri , P. Allen , X. Xu , H. Joshi , Y. Fan , L. V. Titova , P. M. Rao , Nano Lett. 2016, 16, 3463. 27203779 10.1021/acs.nanolett.5b05200

[59] J. Liu , J. Li , M. Shao , M. Wei , J. Mater. Chem. A 2019, 7, 6327.

[60] S. M. Thalluri , L. Bai , C. Lv , Z. Huang , X. Hu , L. Liu , Adv. Sci. 2020, 7, 1902102. 10.1002/advs.201902102 PMC7080548 32195077

[61] X. Wang , P. Huo , Y. Liu , Y. Xiang , C. Jia , Z. Yan , Appl. Catal., A 2021, 616, 118073.

[62] J. Li , N. Wu , Catal. Sci. Technol. 2015, 5, 1360.

[63] X. T. Xu , L. Pan , X. Zhang , L. Wang , J. J. Zou , Adv. Sci. 2019, 6, 1801505. 10.1002/advs.201801505 PMC6343073 30693190

[64] J. B. Pan , B. H. Wang , J. B. Wang , H. Z. Ding , W. Zhou , X. Liu , J. R. Zhang , S. Shen , J. K. Guo , L. Chen , Angew. Chem., Int. Ed. 2021, 133, 1453.

[65] D. E. Schipper , Z. Zhao , A. P. Leitner , L. Xie , F. Qin , M. K. Alam , S. Chen , D. Wang , Z. Ren , Z. Wang , ACS Nano 2017, 11, 4051. 28333437 10.1021/acsnano.7b00704

[66] Y. Zhou , L. Zhang , L. Lin , B. R. Wygant , Y. Liu , Y. Zhu , Y. Zheng , C. B. Mullins , Y. Zhao , X. Zhang , G. Yu , Nano Lett. 2017, 17, 8012. 29185764 10.1021/acs.nanolett.7b04626

[67] F. Li , H. Yang , Q. Zhuo , D. Zhou , X. Wu , P. Zhang , Z. Yao , L. Sun , Angew. Chem., Int. Ed. 2021, 133, 2004. 10.1002/anie.202011069 PMC7894348 33051952

[68] J.‐S. Yang , J.‐J. Wu , Nano Energy 2017, 32, 232.

[69] T. Wang , J. Gong , Sci. China Mater. 2017, 60, 90.

[70] A. Banerjee , B. Mondal , A. Verma , V. R. Satsangi , R. Shrivastav , A. Dey , S. Dass , J. Catal. 2017, 352, 83.

[71] O. Khaselev , A. Bansal , J. Turner , Int. J. Hydrogen Energy 2001, 26, 127.

[72] A. Dutta , A. Naldoni , F. Malara , A. O. Govorov , V. M. Shalaev , A. Boltasseva , Faraday Discuss. 2019, 214, 283. 30821797 10.1039/c8fd00148k

[73] L. Zhou , Y. Yang , J. Zhang , P. M. Rao , ACS Appl. Mater. Interfaces 2017, 9, 11356. 28326767 10.1021/acsami.7b01538

[74] S.‐Y. Chen , J.‐S. Yang , J.‐J. Wu , ACS Appl. Energy Mater. 2018, 1, 2143.

[75] Y. F. Xu , H. S. Rao , B. X. Chen , Y. Lin , H. Y. Chen , D. B. Kuang , C. Y. Su , Adv. Sci. 2015, 2, 1500049. 10.1002/advs.201500049 PMC5115430 27980959

[76] W. Wang , C. Jin , L. Qi , Small 2018, 14, 1801352. 10.1002/smll.201801352 30027578

[77] Z. Wang , T. D. Nguyen , L. P. Yeo , C. K. Tan , L. Gan , A. I. Y. Tok , Small 2020, 16, 1905826. 10.1002/smll.201905826 31916682

[78] B. R. Lee , M. G. Lee , H. Park , T. H. Lee , S. A. Lee , S. S. M. Bhat , C. Kim , S. Lee , H. W. Jang , ACS Appl. Mater. Interfaces 2019, 11, 20004. 31083922 10.1021/acsami.9b03712

[79] T. Jin , P. Diao , Q. Wu , D. Xu , D. Hu , Y. Xie , M. Zhang , Appl. Catal., B 2014, 148, 304.

[80] Z. Ma , K. Song , L. Wang , F. Gao , B. Tang , H. Hou , W. Yang , ACS Appl. Mater. Interfaces 2019, 11, 889. 30560657 10.1021/acsami.8b18261

[81] Y. Wang , W. Tian , L. Chen , F. Cao , J. Guo , L. Li , ACS Appl. Mater. Interfaces 2017, 9, 40235. 29067799 10.1021/acsami.7b11510

[82] Y. K. Hsu , Y. C. Chen , Y. G. Lin , ACS Appl. Mater. Interfaces 2015, 7, 14157. 26053274 10.1021/acsami.5b03921

[83] S. J. A. Moniz , J. Zhu , J. Tang , Adv. Energy Mater. 2014, 4, 1301590.

[84] D. Kim , Z. Zhang , K. Yong , Nanoscale 2018, 10, 20256. 30362492 10.1039/c8nr06630b

[85] L. Wang , J. Han , Y. Wu , Y. Zhang , Q. Zhang , X. Tan , Y. Yang , W. Li , Y. Bu , J.‐P. Ao , Chem. Eng. J. 2019, 368, 710.

[86] Y.‐F. Xu , H.‐S. Rao , X.‐D. Wang , H.‐Y. Chen , D.‐B. Kuang , C.‐Y. Su , J. Mater. Chem. A 2016, 4, 5124.

[87] Z. Bai , X. Yan , Y. Li , Z. Kang , S. Cao , Y. Zhang , Adv. Energy Mater. 2016, 6, 1501459.

[88] Y. Liu , Z. Kang , H. Si , P. Li , S. Cao , S. Liu , Y. Li , S. Zhang , Z. Zhang , Q. Liao , L. Wang , Y. Zhang , Nano Energy 2017, 35, 189.

[89] K. Kim , J. H. Moon , ACS Appl. Mater. Interfaces 2018, 10, 34238. 30265510 10.1021/acsami.8b11241

[90] L. Zhang , E. Reisner , J. J. Baumberg , Energy Environ. Sci. 2014, 7, 1402.

[91] Q. Zeng , Y. Gao , L. Lyu , S. Chang , C. Hu , Nanoscale 2018, 10, 13393. 29995056 10.1039/c8nr03122c

[92] A. Kormányos , E. Kecsenovity , A. Honarfar , T. Pullerits , C. Janáky , Adv. Funct. Mater. 2020, 30, 2002124. 32774199 10.1002/adfm.202002124 PMC7405979

[93] Y. W. Phuan , M. N. Chong , J. D. Ocon , E. S. Chan , Sol. Energy Mater. Sol. Cells 2017, 169, 236.

[94] P. Qiu , F. Li , H. Zhang , S. Wang , Z. Jiang , Y. Chen , Electrochim. Acta 2020, 358, 136847.

[95] J. H. Kim , J. S. Lee , Adv. Mater. 2019, 31, 1806938.

[96] J. Choi , T. Song , J. Kwon , S. Lee , H. Han , N. Roy , C. Terashima , A. Fujishima , U. Paik , S. Pitchaimuthu , Appl. Surf. Sci. 2018, 447, 331.

[97] Q. Jiang , X. Zhang , J. You , Small 2018, 14, 1801154.

[98] S. Annathurai , S. Chidambaram , B. Baskaran , G. P. Venkatesan , J. Inorg. Organomet. P. 2019, 29, 535.

[99] K. Yuan , Q. Cao , H.‐L. Lu , M. Zhong , X. Zheng , H.‐Y. Chen , T. Wang , J.‐J. Delaunay , W. Luo , L. Zhang , Y.‐Y. Wang , Y. Deng , S.‐J. Ding , D. W. Zhang , J. Mater. Chem. A 2017, 5, 14697.

[100] Y. Liu , B. R. Wygant , K. Kawashima , O. Mabayoje , T. E. Hong , S.‐G. Lee , J. Lin , J.‐H. Kim , K. Yubuta , W. Li , Appl. Catal., B 2019, 245, 227.

[101] L. Yao , Y. Liu , H.‐H. Cho , M. Xia , A. Sekar , B. P. Darwich , R. A. Wells , J.‐H. Yum , D. Ren , M. Grätzel , Energy Environ. Sci. 2021, 14, 3141.

[102] P. Wei , K. Lin , D. Meng , T. Xie , Y. Na , ChemSusChem 2018, 11, 1746. 29700973 10.1002/cssc.201800705

[103] J. Resasco , H. Zhang , N. Kornienko , N. Becknell , H. Lee , J. Guo , A. L. Briseno , P. Yang , ACS Cent. Sci. 2016, 2, 80. 27163032 10.1021/acscentsci.5b00402 PMC4827543

[104] M. G. Lee , D. H. Kim , W. Sohn , C. W. Moon , H. Park , S. Lee , H. W. Jang , Nano Energy 2016, 28, 250.

[105] A. Hankin , F. E. Bedoya‐Lora , J. C. Alexander , A. Regoutz , G. H. Kelsall , J. Mater. Chem. A 2019, 7, 26162.

[106] S. K. Karuturi , J. Luo , C. Cheng , L. Liu , L. T. Su , A. I. Tok , H. J. Fan , Adv. Mater. 2012, 24, 4157. 22641295 10.1002/adma.201104428

[107] C. Cheng , S. K. Karuturi , L. Liu , J. Liu , H. Li , L. T. Su , A. I. Tok , H. J. Fan , Small 2012, 8, 37. 22009604 10.1002/smll.201101660

[108] L. Ran , S. Qiu , P. Zhai , Z. Li , J. Gao , X. Zhang , B. Zhang , C. Wang , L. Sun , J. Hou , J. Am. Chem. Soc. 2021, 143, 7402. 33961743 10.1021/jacs.1c00946

[109] X. Zhang , S. John , Opt. Express 2021, 29, 22376. 34266003 10.1364/OE.427218

[110] Y. Chen , W. Zheng , S. Murcia‐López , F. Lv , J. R. Morante , L. Vayssieres , C. Burda , J. Mater. Chem. C 2021, 9, 3726.

[111] S. Zhou , R. Tang , L. Zhang , L. Yin , Electrochim. Acta 2017, 248, 593.

[112] Y. Zeng , T. Yang , C. Li , A. Xie , S. Li , M. Zhang , Y. Shen , Appl. Catal., B 2019, 245, 469.

[113] X. Zhang , S. John , J. Mater. Chem. A 2020, 8, 18974.

[114] M. Curti , J. Schneider , D. W. Bahnemann , C. B. Mendive , J. Phys. Chem. Lett. 2015, 6, 3903. 26722891 10.1021/acs.jpclett.5b01353

[115] Y. Gun , G. Y. Song , V. H. Quy , J. Heo , H. Lee , K. S. Ahn , S. H. Kang , ACS Appl. Mater. Interfaces 2015, 7, 20292. 26322646 10.1021/acsami.5b05914

[116] I. Kondofersky , H. K. Dunn , A. Muller , B. Mandlmeier , J. M. Feckl , D. Fattakhova‐Rohlfing , C. Scheu , L. M. Peter , T. Bein , ACS Appl. Mater. Interfaces 2015, 7, 4623. 25562687 10.1021/am5078667

[117] J. Yang , C. Bao , T. Yu , Y. Hu , W. Luo , W. Zhu , G. Fu , Z. Li , H. Gao , F. Li , Z. Zou , ACS Appl. Mater. Interfaces 2015, 7, 26482. 26565922 10.1021/acsami.5b07470

[118] X. Li , J. Wang , InfoMat 2020, 2, 3.

[119] Z. He , J. Zhang , X. Li , S. Guan , M. Dai , S. Wang , Small 2020, 16, 2005051. 10.1002/smll.202005051 33103848

[120] J.‐R. Ding , K.‐S. Kim , Chem. Eng. J. 2018, 334, 1650.

[121] S. Li , Z. Wang , Y. Yang , J. Li , C. Jin , Micro Nano Lett. 2020, 15, 226.

[122] M. Stefik , M. Cornuz , N. Mathews , T. Hisatomi , S. Mhaisalkar , M. Gratzel , Nano Lett. 2012, 12, 5431. 22974097 10.1021/nl303101n

[123] L. Meng , D. Rao , W. Tian , F. Cao , X. Yan , L. Li , Angew. Chem., Int. Ed. 2018, 57, 16882. 10.1002/anie.201811632 30371007

[124] Z. Zhang , C. Gao , Y. Li , W. Han , W. Fu , Y. He , E. Xie , Nano Energy 2016, 30, 892.

[125] S. Ju , H. Kang , J. Jun , S. Son , J. Park , W. Kim , H. Lee , Small 2021, 17, 2006558. 10.1002/smll.202006558 33864345

[126] J. H. Strait , P. A. George , M. Levendorf , M. Blood‐Forsythe , F. Rana , J. Park , Nano Lett. 2009, 9, 2967. 19594164 10.1021/nl901373j

[127] J. LaForge , T. Cocker , A. Beaudry , K. Cui , R. Tucker , M. Taschuk , F. Hegmann , M. Brett , Nanotechnology 2013, 25, 035701. 24346484 10.1088/0957-4484/25/3/035701

[128] X. Huai , L. Girardi , R. Lu , S. Gao , Y. Zhao , Y. Ling , G. A. Rizzi , G. Granozzi , Z. Zhang , Nano Energy 2019, 65, 104020.

[129] C. J. Raj , S. Karthick , K. Hemalatha , S.‐K. Kim , B. C. Kim , K.‐H. Yu , H.‐J. Kim , Appl. Phys. A 2014, 116, 811.

[130] I. S. Cho , Z. Chen , A. J. Forman , D. R. Kim , P. M. Rao , T. F. Jaramillo , X. Zheng , Nano Lett. 2011, 11, 4978. 21999403 10.1021/nl2029392

[131] M. Fang , G. Dong , R. Wei , J. C. Ho , Adv. Energy Mater. 2017, 7, 1700559.

[132] L. Cai , J. Zhao , H. Li , J. Park , I. S. Cho , H. S. Han , X. Zheng , ACS Energy Lett. 2016, 1, 624.

[133] X. Li , M. Puttaswamy , Z. Wang , C. Kei Tan , A. C. Grimsdale , N. P. Kherani , A. I. Y. Tok , Appl. Surf. Sci. 2017, 422, 536.

[134] D. Peeters , A. Sadlo , K. Lowjaga , O. Mendoza Reyes , L. Wang , L. Mai , M. Gebhard , D. Rogalla , H. W. Becker , I. Giner , Adv. Mater. Interfaces 2017, 4, 1700155.

[135] L. Wen , M. Zhou , C. Wang , Y. Mi , Y. Lei , Adv. Energy Mater. 2016, 6, 1600468.

[136] M. Leskela , M. Ritala , Angew. Chem., Int. Ed. 2003, 42, 5548. 10.1002/anie.200301652 14639717

[137] R. W. Johnson , A. Hultqvist , S. F. Bent , Mater. Today 2014, 17, 236.

[138] Y. H. Xiao , W. Tian , S. Jin , Z. G. Gu , J. Zhang , Small 2020, 16, 2005111. 10.1002/smll.202005111 33078581

[139] W. Zhang , J. Huang , J. Xu , M. Han , D. Su , N. Wu , C. Zhang , A. Xu , C. Zhan , Adv. Energy Mater. 2020, 10, 2001436.

[140] L. Li , K. Wang , J. Pei , Z. Qin , Y. Hu , Q. Dong , G. Chen , Adv. Mater. Interfaces 2021, 8, 2001847.

[141] S. D. Seo , S. Yu , S. Park , D. W. Kim , Small 2020, 16, 2004806.

